# Demonstration of *Escherichia coli* Inactivation in Sterile Physiological Saline under High Pressure (HP) Phase Transition Conditions and Analysis of Probable Contribution of HP Metastable Positions Using Model Solutions and Apple Juice

**DOI:** 10.3390/foods11081080

**Published:** 2022-04-08

**Authors:** Ting Xiao, Yifan Li, Lihui Hu, Pengcheng Nie, Hosahalli S. Ramaswamy, Yong Yu

**Affiliations:** 1 College of Biosystems Engineering and Food Science, Zhejiang University, 866 Yuhangtang Road, Hangzhou 310058, China; tinyaxiao@zju.edu.cn (T.X.); yifanli@zju.edu.cn (Y.L.); xytsmf474@163.com (L.H.); pcn@zju.edu.cn (P.N.); 2 Key Laboratory of Equipment and Informatization in Environment Controlled Agriculture, Ministry of Agriculture, 866 Yuhangtang Road, Hangzhou 310058, China; 3 Department of Food Science and Agricultural Chemistry, McGill University, 21111 Lakeshore Road, St-Anne-de-Bellevue, QC H9X 3V9, Canada

**Keywords:** high-pressure-subzero-temperature, phase transition, fruit and vegetable system models, microbial destruction, phase transition position

## Abstract

It was demonstrated that the inactivation of high pressure (HP) treatment on *Escherichia coli* survival in sterile physiological saline (SPS) was influenced by the treatment conditions: unfrozen, frozen-thawed and fully frozen (phase transition). In order to probe the enhanced phase transition microbial destruction, vibration effects of phase transition position were created and discussed. Test samples were placed in HP chamber for treatment (150/240/330 MPa, no holding time) at room temperature and a special cooling device was used to maintain the phase transition conditions. Results showed that the phase transition from ice I to ice III of frozen SPS could be realized based on the cooling of a 20% sodium chloride solution. HP treatment under fully frozen conditions produced the best lethal effect compared to unfrozen and freeze-thaw samples. Vibration tests were carried out by using model solutions and apple juice to explore the behavior of phase transition. A synchronous and advance phase transition of internal apple juice was realized, respectively, by using pure water and 5% sodium chloride solution as external vibration sources, and the advance phase transitions of external pure water were realized by using 5% sodium chloride solution and 5% glucose solution as internal vibration sources.

## 1. Introduction

High pressure (HP) processing is becoming a high-tech pasteurization/sterilization technology which is attracting much attention all over the world because it overcomes the thermal damage to quality and nutrients in food that characterizes conventional thermal processing [1,2,3,4]. In recent years, the deficiency of HP processing has gradually emerged in some areas. The main disadvantage of HP processing is that it has limited commercial sterilization ability for pathogenic spore forming bacteria which have strong pressure resistance [5]. Considering the processing parameters, the main factors affecting the inactivation level are pressure level, pressure holding time and temperature; the higher levels of these three parameters create the inactivation effect [6,7,8]. However, in practical commercial application, while considering the safety and nutrition of food itself, one should also take into account the technical cost to achieve the low cost and high efficiency of HP processing. Therefore, in terms of operation of the HP equipment, the trend with respect to the three parameters is generally the opposite: the lower the better. Huang et al. [9] found that in order to reduce the number of *Escherichia coli* O157:H7 in carambola juice to a safe level, a pressure of up to 600 MPa needed to be applied. From the perspective of economic cost and safety guarantee demand, the pressure of HP processing in commercial application should not be so great. Therefore, researchers have begun to shift the focus of HP applications using the traditional and other variables in other ways.

At present, the synergistic treatment of HP and low temperature is considered to be effective for pasteurization methods [10,11]. Theoretically, the research on the synergistic treatment of HP and temperature includes at least two angles: one is the synergy between HP and high temperature (HPHT), and the other is the synergy between high pressure and low temperature (HPLT). Among them, the combination of HP and high temperature achieves a higher bactericidal effect on microbial spores than when the two are used independently [10,12]. Although the synergistic high temperature is not as high as that of thermal pasteurization, it also gives up some heat sensitive quality of food under the condition of ensuring the inactivation effect [13,14]. Sometimes, in order to pursue a better commercial pasteurization effect, the synergistic high temperature is close to that of conventional thermal pasteurization. This makes the research of this method gradually deviate from the field of non-thermal processing and gradually lose the "quality assurance" advantage of HP processing. In contrast, the synergy between high pressure and low temperature shows greater potential for HP pasteurization [15,16,17,18].

**Figure 1:**
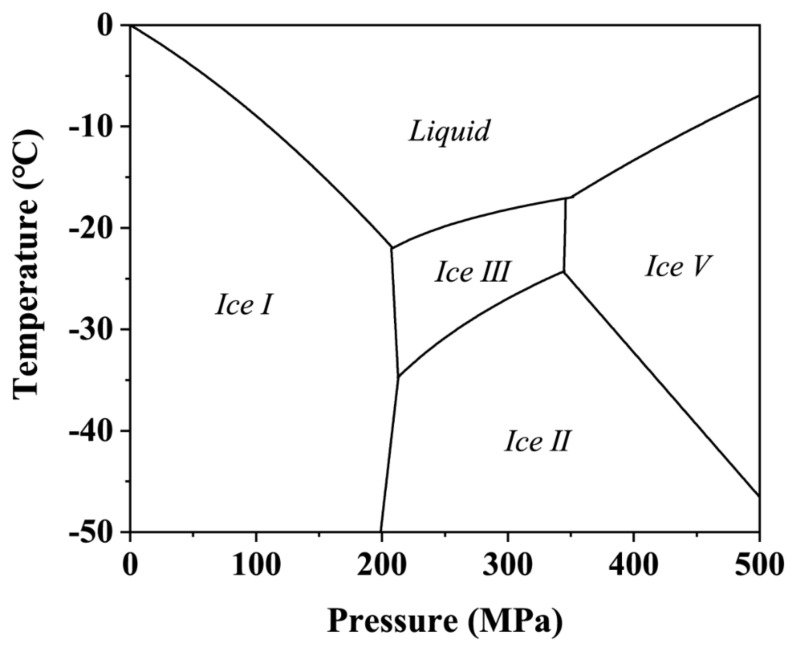
Phase diagram of pure water under high pressure based on data reported by Bridgman [16].

Under high pressure and low temperatures, water can form different ice crystals and undergo different solid-solid phase transitions [15]. Under the conventional low temperature and pressure conditions, at least four ice crystal forms (Figure 1) with different densities have been shown to exist: ice I, ice II, ice III and ice V [15,16]. Ice III is the easiest to form next to ice I [15], so the phase transition from ice I to ice III is also easy to achieve. Phase change microbial destruction makes use of the mechanical stress generated by the change in volume during the phase transformation to ice crystals to different forms [17]. Several studies focused on HPLT have some shortcomings: (1) only few equipment can realize freezing under high pressure, but the chamber size is small, the processing capacity is limited, the cooling speed is slow, and the efficiency is low [18,19], and commercialization potential is practically nil; (2) only the temperature of the pressure transmission medium can be detected, which will not reflect the real temperature of the sample during HP treatment [20]. These high equipment requirements and the difficulty in monitoring sample temperatures limit phase change inactivation studies. In addition, the phase change pressure is an important parameter in the phase change inactivation, taking advantage of the phase change position. Due to existence of the metastable state, the phase change position is unstable [21,22,23]. Exploration of the influence of phase change position will help to better understand the phase change kinetics. Due to equipment and temperature monitoring limitations, and lack of knowledge of the influence of phase change position, available scientific information in this area is very scarce.

A custom developed laboratory-designed self-cooling device was used in this study. This cooling system achieves automatic cooling in the HP chamber set-up without the necessity for any external temperature control system on the traditional high-pressure apparatus. Thus, it can be employed in any existing high pressure equipment operating at room temperature. Compared with the previous complex and expensive HPLT equipment, this way to realize high-pressure and low temperature is both convenient and economical. Li et al. [6] tested the cooling efficiency of the device and reported that it only took 1.5 min for the milk center temperature to change from −21 °C to −40 °C The volume of the sample was flexible, and it would not be limited like the small volume pressure chambers in previous studies [18,19].

Sodium chloride and glucose exist in many real foods, including fruits and vegetables [24]. Their phase transitions are similar to those of a real food system, so they can be used as models of real food. Therefore, these were selected as models to simulate the fruit and vegetable system for phase-change analysis. The pH value of food is one of the important factors affecting microbial inactivation. In general, too high or too low a pH increases the sensitivity of microorganisms, making them less viable and thus more susceptible to be inactivated under HP [25]. Therefore, 0.85% sodium chloride solution (sterile physiological saline, SPS) with a neutral pH value was selected for the microbial destruction studies to exclude the influence of pH itself on microbial inactivation. In this case, the microbial survival ability under HP might be stronger [26], which is more conducive for testing the low acid HPLT destruction ability, avoiding the final inactivation effect of the combination of HPLT and pH value.

Therefore, the purpose of this study was to use a laboratory-designed self-cooling device to: (1) obtain the temperature and phase transition information for establishing an HPLT process and demonstrate the inactivation of *Escherichia coli* in sterile physiological saline by HP treatment given to samples in three different forms: unfrozen, frozen-thawed and fully frozen (phase transition); (2) carry out vibration tests by using model systems as possible link for the inactivation and the law of phase transition.

## 2. Materials and Methods

### 2.1. Cooling System

**Figure 2:**
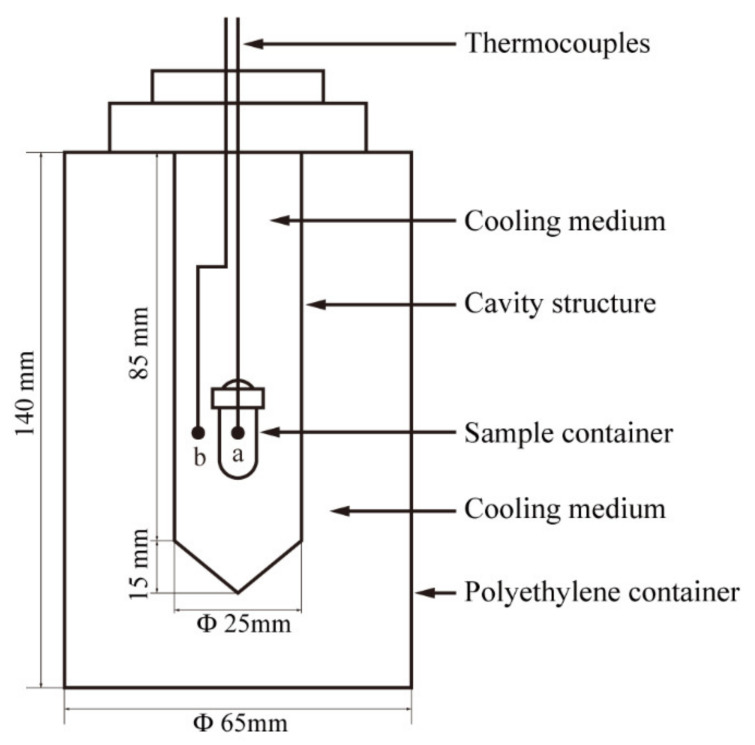
Schematic diagram of cooling system for the *E. coli* inoculated SPS.

A laboratory-designed self-cooling device (Figure 2) was used in this study. This cooling system can realize automatic cooling in the HP chamber without assembling an additional temperature control system on the traditional HP apparatus, so as to ensure the operation of HPLT studies in any commercial HP equipment. The main body of the cooling system is a cylindrical polyethylene container (65 mm in diameter and 140 mm in height). Typically, an empty 50 mL centrifuge tube is positioned at the top of the polyethylene container filled with the cooling medium (20% sodium chloride solution), and then the polyethylene bottle is placed in a −30 °C freezer for 48 h. After complete freezing, the centrifugal tube is pulled out to form a cavity to the center of the bottle. Afterwards, a sample container (the volume varied with the specific experiment) is placed in the cavity, and the cooling medium is poured to fill the remaining space. Two K-type thermocouples (OMEGA Engineering, Stamford, CT, USA) connected to a data collector (34970A, Agilent Technologies GMBH, Germany) are used, one inserted into the center of the sample container (a in Figure 2) to measure the sample temperature, another to record the temperature of the cooling medium (b in Figure 2), and then the bottle is closed. Finally, the polyethylene container containing the sample is vacuum-packed in a flexible polyethylene bag and frozen at −30 °C for 24 h to obtain the cooling system capable of achieving low temperatures under high pressure. In the cooling system, the initial temperature of the cooling medium and the sample was maintained at −30 °C.

### 2.2. Escherichia coli Strain and Culture Preparation

Freeze dried cultures of *E. coli* strains ATCC 25922 (CGMCC 1.2385) were used and purchased from China General Microbiological Culture Collection Center (CGMCC, Beijing, China). For the revival of freeze-dried strains, sterile nutrient broth (Sinopharm Chemical Reagent Co., Ltd., Shanghai, China) was used as liquid medium for initial activation at 37 °C for 24 h, and then the resuscitated cultures were inoculated to a sterile nutrient agar (Sinopharm Chemical Reagent Co., Ltd., Shanghai, China) slant and incubated at 37 °C for 24 h before being stored at 4 °C. In order to maintain the viability of strains, we made new sterile nutrient agar slant every month, and then used inoculant ring to pick out several bacterial strains from the old nutrient agar slant and transferred them to the new nutrient agar slant in the form of a scribed curve. They were cultured at 37 °C for 24 h and then stored at 4 °C. To prepare the working cultures, several loops of the strain were scraped from the slant of the nutrient agar, and then inoculated into 50 mL nutrient broth amd placed in a 37 °C thermostatic oscillator bath (HZ-9211KB, Taicang Hualida Experimental Instrument Co., Ltd., Suzhou, China) for 24 h at a shake speed of 150 rpm. On the second day, several loops of *E. coli* suspension in the above nutrient broth were re-inoculated in to 150 mL nutrient broth and incubated at 37 °C for 24 h to obtain the inoculum for testing.

### 2.3. Sample Preparation

To prepare the sterile physiological saline (SPS), 8.5 g sodium chloride crystals were dissolved in 1000 mL distilled water and then autoclaved at 121 °C for 15 min. The inoculum obtained in Section 3.1 was placed in a 50 mL round-bottom centrifuge tube, centrifuged (5810R, Eppendorf AG, Hamburg, Germany) at 3200 g for 5 min at 20 °C, the *E. coli* pellets were obtained, and the supernatant was discarded. After washing the pellets several times with SPS, the *E. coli* cells suspended in SPS were divided into five parts after shaking evenly by an oscillator. The first part used for plating on brain-heart infusion agar (BHIA, Beijing Coolaber Science & Technology Co. Ltd., Beijing, China) and the initial population was counted as about 10^8^ CFU/mL. The other four parts were packed in 9 mL sample containers, one of which was used as an untreated frozen sample, and the other three were used for HP treatment.

### 2.4. High Pressure Treatment

**Figure 3:**
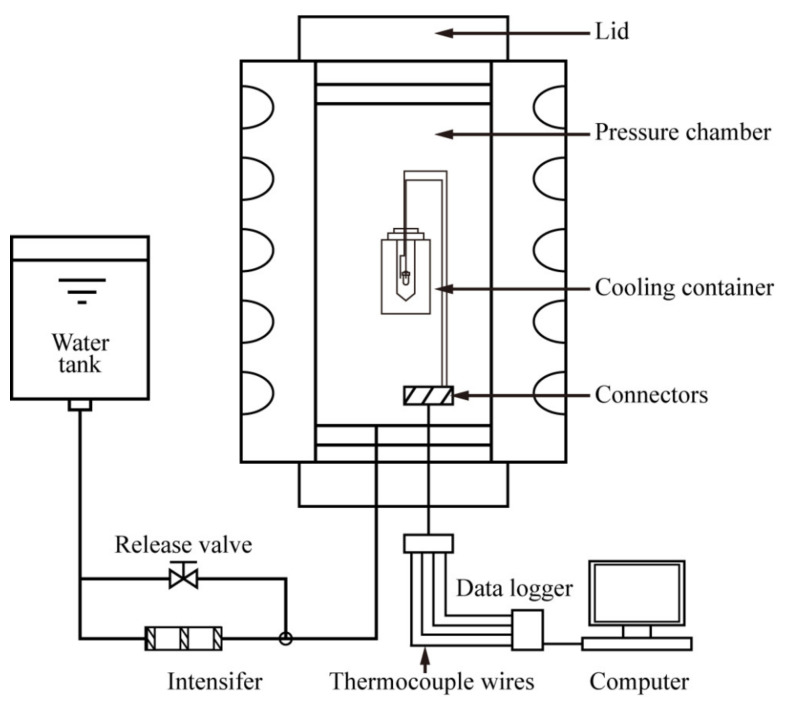
Schematic diagram of the high-pressure apparatus.

As shown in Figure 3, the high pressure (HP) treatment was given in a 5 L chamber HP unit (UHPF-750, Baotou Kefa High Pressure Technology Co., Ltd., Baotou, China). The pressurization medium was pure water, and the pressure chamber was maintained at 20 °C (room temperature) before pressurization. Three pressure levels of 150, 240 and 330 MPa were selected for the study, and the pressure holding time was 0 s. The lethal effect of HP on *E. coli* in three different formats: unfrozen state, frozen-thawed state and the fully frozen (phase transition) state. The three prepared samples reserved for HP experiment in Section 2.3 were used for treatment in the three different states.

The unfrozen-state experiment meant the samples obtained in Section 2.3 were vacuum-packed in flexible polyethylene bags and directly placed into the pressure chamber for HP treatment. The frozen-thawed-state experiment meant that the samples were placed in a −30 °C freezer for 24 h, and then the frozen samples were completely thawed in water at 4 °C and vacuum-packed in flexible polyethylene bags and treated with HP. The frozen-state experiment was carried out based on the cooling system mentioned in Section 2.1, with minor changes in the operating procedures. After obtaining the cavity structure in the middle of the frozen cooling medium, the sample container and 10 mL anhydrous ethanol were put into a heat-sealing bag, and then placed into the cavity. After filling the remaining gap with the cooling medium, the cooling medium container was frozen at −30 °C for 24 h and then subjected to HP tests. The purpose of adding anhydrous ethanol around the sample was to create a liquid pressure transfer environment for the sample and avoid damage to the sample container caused by a solid pressure transfer environment of frozen cooling medium. After HP treatment, the samples before colony counting were stored in a 4 °C freezer, and the counting time was no more than 2 h. All experiments were carried out in triplicate.

### 2.5. Enumeration of E. coli

Brain-heart infusion agar (BHIA, Beijing Coolaber Science & Technology Co. Ltd., Beijing, China) was selected to evaluate the lethal effect of HP treatment on *E. coli*. The untreated frozen samples and pressure-treated samples were transferred to a sterile ultra-clean platform and homogenized for 10 s with a stomacher to ensure the uniform distribution of *E. coli*. Sterile physiological saline (0.85% *w*/*v* NaCl) was used for serial dilution of samples. After the BHIA was cooled and solidified, the plates were turned over and incubated at 37 °C for 48 h. Colony count was taken after two days.

### 2.6. Evaluation of the Effect of Vibration on Phase Transition Position

In order to further explore the factors affecting the phase transition position of food, the influence of vibration factors on the phase transition position of ice I to ice III of water in apple juice and model solutions was investigated. In the vibration test, the composition and operation steps of the cooling system were somewhat different from that described in Section 2.1, and the specific experimental design is shown in Table 1. According to the different experimental methods, it was divided into internal vibration experiment and external vibration experiment. The specific principles would be described in detail in Section 3.3.

For internal vibration experiments, the cooling medium container with the 50 mL centrifugal tube was frozen for 48 h, and the centrifuge tube was not pulled out but left as the sample container. Then the sample was poured into the 50 mL centrifuge tube, and the cap was twisted on, and the sample was frozen at −30 °C for 24 h. For external vibration experiment, a 4 mL sample container was placed into the cavity structure, and cold pure water was poured to fill the remaining gap. The reason for pouring cold pure water was to increase the volume change during phase transformation and increase vibration intensity. The mechanism can be explained as follows: when the phase transition from ice I to ice III occurs in the solution, it is water as a solvent that causes the volume change. Compared with 5% glucose solution and 5% sodium chloride solution, there is no solute in pure water. Therefore, when the phase transition from ice I to ice III occurs between the same volume of solution and pure water, the content of solvent water in the solution is less than that of pure water due to the existence of solute. Thus, the volume change of pure water is also the largest due to the largest volume during phase transition. After screwing on the cap, the sample was put into the −30 °C freezer for 24 h. The rest of the cooling system composition and operation procedures were exactly as described in Section 2.1. All experiments were repeated at least 3 times.

### 2.7. Statistical Analysis

SPSS (24.0, Statistical Package for the Social Sciences, SPSS Inc., Armonk, NY, USA) software was used for one-way analysis of variance (ANOVA) and the differences in average values were compared (Duncan’s test, *p* < 0.05).

## 3. Results and Discussion

### 3.1. Temperature and Phase Transition Behavior in Frozen SPS during HP Treatment

**Figure 4:**
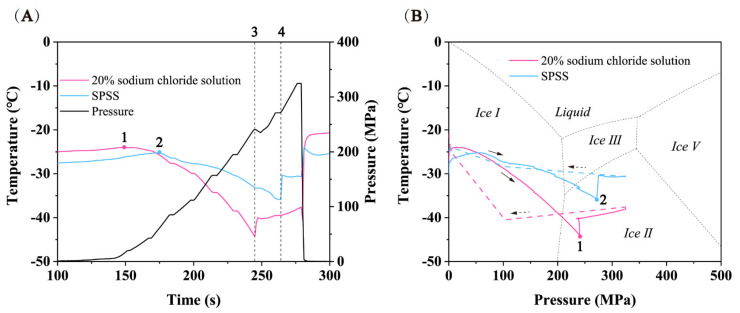
Temperature and pressure paths of frozen inoculated SSPS cooled by 20% sodium chloride solution. (**A**) Temperature and pressure curves over time. (**B**) Temperature-pressure profile superimposed on the phase diagram of water. (—) pressure boost process, (---) pressure relief process.

Figure 4 shows the temperature and pressure profiles of frozen SPS cooled by 20% sodium chloride solution and pressurized to 330 MPa and depressurized immediately (zero holding time). The freezing temperature of 20% sodium chloride and sterile physiological saline under atmospheric pressure was −21.01 °C and −4.58 °C, respectively. When the cooling system was removed from the freezer, the initial temperature of the sample and the cooling medium was −30 °C. Before the pressure began to rise, the temperature of the cooling medium and the sample rose to −25.03 °C and −27.52 °C, respectively. This may be caused by the heat exchange between the cooling system and the surrounding air (20 °C) and the water in the pressure chamber (20 °C). Because the cooling medium was in direct contact with the outside air and liquid water, and the inner sample was protected by the outer cooling medium, temperature rise of the cooling medium was greater than for the sample.

As the pressure started to rise, the temperature of cooling medium and the frozen SPS increased to −24.01 °C and −25.22 °C, respectively. This may be caused by adiabatic compression and heat exchange with the pressure transfer medium, but they both still remained in the ice I state. As the temperature increased, the cooling medium soon reached the ice I-water phase transition point (Figure 4A(1)) and began melting. At this point, the pressure was around 15 MPa, and then the cooling medium temperature started to decline along its own ice I-water phase transition equilibrium curve. Due to the presence of anhydrous ethanol between the sample and the cooling medium, the temperature change had a certain lag, which slowed down the temperature change in the sample [27]. As a result, the sample temperature did not immediately decrease along with the cooling medium temperature, as reported in earlier studies [6,16,28]. The sample temperature began to decrease when pressure reached ~60 MPa (Figure 4A(2)).The temperature drop in the sample could be affected by three processes: (1) the frozen cooling medium (20% sodium chloride solution) dropped along its own ice I-water phase transition equilibrium curve, and kept melting and absorbing heat in this process, resulting in the temperature drop of the internal sample; (2) the temperature of the frozen cooling medium was lower than that of the frozen sample, and the temperature difference between the two produced heat exchange, thus reducing the sample temperature further; (3) adiabatic compression led to a small temperature rise. The above three processes eventually led to a larger drop in sample temperature overcoming the adiabatic increase, resulting in an overall decline in the sample temperature. The same phenomenon has been observed in earlier studies as well [6,28].

Surprisingly, when the pressure increased to the triple point of liquid-ice I to ice III (210 MPa), neither the sample nor the cooling medium underwent the phase transition from ice I to ice III. Based on the phase diagram of pure water [15], the phase transition from ice I to ice III should have occurred at 210 MPa, but unexpected results and metastable regions were observed as the pressure continued to increase. For cooling medium, the frozen 20% sodium chloride solution continued to descend along its own phase transition extension curve of ice I to water, although, as shown in Figure 4B, it entered the ice II region of the pure water phase diagram. This was because the freezing point of 20% sodium chloride solution (−21.01 °C) was much lower than that of the pure water [6] and its own phase diagram moved downward relative to the pure water phase diagram. Therefore, in fact, the cooling medium entered the metastable region in the domain of ice III. Previous studies have demonstrated that the extension line of ice I-water phase transition curve existed in the thermodynamic stable domain of ice III, and metastable ice I and metastable water also existed [18,22]. Consequently, it can be inferred that the cooling medium was composed of metastable ice I and metastable water in this area. When the pressure was increased to 240 MPa (Figure 4A(3),B(1)), the temperature suddenly increased from −44.2 °C to −40.0 °C. The reason for this phenomenon may be that the frozen 20% sodium chloride solution underwent the phase transitions from metastable ice I to ice III (endothermic reaction) and metastable water re-crystallization to ice III (exothermic reaction). The two processes eventually led to the release of heat and a temperature rise. Afterward, the temperature changed with the phase transition curve of ice III-water. In addition, the phase change process made the pressure drop by 6 MPa. Since the density of ice III is 1.14 g/cm^3^, which is larger than that of ice I (0.92 g/cm^3^) and liquid water (1 g/cm^3^), the phase transformation of metastable ice I and metastable water to ice III was a process of volume reduction on the whole. Consequently, the cooling medium with a volume of nearly 440 mL produced a volume change during the phase transition, resulting in a pressure drop of ~6 MPa. For the frozen sample, the frozen SSPS entered the metastable zone of ice III and ice II successively after the pressure exceeded 210 MPa. As reported in earlier studies, metastable ice I was observed in the ice II region, and the nucleation of ice III could be realized in the ice II region [29]. Hence, when the pressure reached ~270 MPa, the frozen SSPS reached the critical point of phase transition, and the phase transition from metastable ice I and metastable water to ice III occurred (Figure 4A(4),B(2)), accompanied by an increase in temperature from −35.9 °C to −30.4 °C. The corresponding pressure drop phenomenon was not observed when the sample underwent phase transition. This may be due to the fact that the frozen sample was only 9 mL and the volume change generated during the phase transition was too small to cause a measurable pressure drop. Depressurization was carried out when the pressure reached 330 MPa, and the phase transition from ice III to metastable water and then to ice I occurred in the sample and cooling medium.

The existence of metastable ice I and metastable water in the thermodynamically stable regions of ice III and ice II made the transition from ice I to ice III very unstable, and the phase transition range may differ greatly (more than 100 MPa) [29]. Therefore, when conducting the experiment of phase transition microbial destruction, the rough pressure range of ice I-ice III phase transition (240 MPa~330 MPa) needed to be determined by pressure and temperature curves first, and then the treated pressure can be determined. Through the verification of repetitive experiments, three treated pressures (150, 240, 330 MPa) were finally determined, so as to explore the effect of ice I-ice III phase transition on the reduction of *Escherichia coli* cells. As the extension of the holding time did not significantly improve the destruction effect of phase change [19], experiments were carried out with zero holding time.

### 3.2. Demonstration of HP Inactivation of E. coli in Samples Treated in Three Formats

Table 2 shows the lethality extent of *E. coli* cells in unfrozen, freeze-thawed and frozen SPS conditions after different HP treatments. The initial concentration of *E. coli* before freezing was 1.4 × 10^8^ CFU/mL. In the process of atmospheric freezing, the formation and growth of ice crystals led to a small destruction of bacterial cell membrane, resulting from damage to microbial cells [30]. The inactivation of *E. coli* caused by freezing observed in this experiment was relatively very small, only 0.14 log, which may be due to the rapid cooling rate which offers better protection against cellular damage due to the associated small size ice crystals. Similar results were obtained previously by Zhu et al. [31] who observed only 0.1 log reduction for *E. coli* in frozen carrot juice after freezing. When the freezing speed was slow, the damage of bacterial cells could be higher [32].

For unfrozen samples, the factor affecting *E. coli* reduction was only the pressure level. The inactivation effect was very small at 150 MPa and 240 MPa: only 0.01 log and 0.25 log, respectively, which also indicated the strong pressure resistance of *E. coli* in SPS. When the pressure was 330 MPa, the inactivation *E. coli* increased to 1.14 log, a small step increase. Obviously, for unfrozen samples, 300 MPa HP treatment did not offer a significant advantage in inactivation of *E. coli*. The lethal effect of high-pressure treatment on *E. coli* cells in unfrozen SPS were similar to some previous studies [6,16,30,31].

For the frozen thawed samples after treatment with high pressure, *E. coli* destruction was not only dependent on the pressure, but also by the freezing process before pressurization. Although freezing alone resulted in only 0.14 log reduction in *E. coli*, after 24 h of freezing, some of the surviving *E. coli* were likely injured by the freezing treatment due to ice crystal formation but could recover in the brain heart medium, so an overall decrease in population was not shown. These bacteria injured cells could possibly be inactivated under pressure. As a result, high pressure treatment of frozen-thawed samples resulted in a much higher cell destruction than that of unfrozen samples. The destruction of *E. coli* at 150, 240 and 330 MPa were 1.31, 1.54 and 2.92 log, respectively. The microbial reduction at 150 MPa was similar to the lethal effect caused at 330 MPa treatment of unfrozen samples, implying that to achieve the same inactivation effect, the pressure required for frozen-thawed samples would be much lower than that of unfrozen samples.

For the pre-frozen samples, the inactivation effect of HP treatment on *E. coli* should be considered from three aspects: (1) the freezing process before compression, (2) the synergistic effect of high pressure and freezing during high pressure treatment and (3) phase transition from ice I to ice III. The freezing process before pressurization made some *E. coli* cells injured, and the injured cells were more likely to be destroyed during pressurization. The synergistic effect of high pressure and freezing can be understood as follows: during the HP treatment of frozen samples, *E. coli* cells in SPS remained in the frozen state, and the pressure resistance was significantly lower than that of *E. coli* in a liquid state; at the same time, the samples located within the cooling system were subjected to additional pressure created by the solid cooling medium, and hence *E. coli* cells were subjected to a greater mechanical stress. Under the combined action of the two, the lethal effect of high-pressure treatment of frozen *E. coli* would be higher than that of freezing-thawed *E. coli* and unfrozen *E. coli* samples. The destruction effect achieved at 150 MPa was a little more than the lethal effect of frozen-thawed samples treated at 240 MPa, which would result in reduced operating pressure and costs from the point of view of commercial application. From 240 MPa to 330 MPa, the bactericidal effect was significantly improved by 1.39 log. According to the temperature and pressure curves in Section 2.1, 240~330 MPa was the phase change interval from ice I to ice III, and the inactivation level of *E. coli* before and after phase transition had a significant difference (*p* < 0.05), indicating that the phase transition from ice I to ice III was an important factor affecting the inactivation of *E. coli*. Under the condition of freezing, the rigidity of bacterial cell membrane and cell wall can increase [33], and the phase transitions would produce gaps on the bacterial cell membrane [6], thus destroying the integrity of the cell membrane and ultimately led to cell death.

Comparing the destruction results of *E. coli* under the same pressure and different states, there was no doubt that the inactivation level of *E. coli* in frozen treated samples was the highest, followed by the inactivation of *E. coli* HP treated frozen-thawed sample, and the lethality of *E. coli* in unfrozen samples was the least. With the increase of pressure, the *E. coli* survivors in various states all gradually decreased, and high-pressure treatment of frozen samples had the best bactericidal effect. Although the data related with effect of phase transition of ice under high pressure condition on inactivation of *E. coli* was smaller than those in many other studies [16,29], this was probably due to the physical and chemical properties of food media, especially the pH value. When the pH value is close to neutral, it is more conducive to the growth and survival of microorganisms, which makes microorganisms less likely to be inactivated under HP. In this study, sterile physiological saline with pH value close to neutral was selected as the medium, which may lead to the final microbial inactivation data being smaller than that in other studies. Zhu et al. [31] selected carrot juice with a pH value of 6.5 for phase change microbial destruction experiment. As the low acidic condition of carrot juice was conducive to the survival of pathogenic microorganisms, *E. coli* was killed by about 3 logs at 400 MPa. Similarly, Li et al. [6] chose milk with a pH value of 6.65, and the HPLT phase change of 400 MPa killed 3.43 log of *E. coli*. In our experiment, the HPLT phase transition at 330 MPa killed 3.68 log of *E. coli* cells, which was relatively small compared with the data of phase transition microbial inactivation in high-acid medium, but relatively reasonable compared with the data of phase transition microbial destruction in food medium with a pH value close to neutral.

### 3.3. Evaluation of the Effect of Vibration on Phase Transition Position

According to the temperature and pressure profiles discussed in Section 3.1, it was clear that there existed a region in the thermodynamic stable domain of ice III in which both metastable ice I and metastable water could co-exist. Some studies have also confirmed these observations [21,22,23]. Due to the metastable ice I in the ice III region, the phase transition from ice I to ice III cannot only depend on the theoretical pressure and temperature conditions, and the metastable characteristics of ice crystals also need to be considered in coordination. In other words, the phase transition conditions from ice I to ice III do not follow a fixed pressure and temperature values, and the metastable water under high pressure will affect the overall phase transition position of food. Therefore, it is considered essential to further evaluate the phase transition position from ice I to ice III.

As the name suggests, the metastable phenomenon is unstable and can be sensitive to external disturbances. Therefore, the vibration source might affect the metastable state, making the metastable limits easier to be broken/altered, and change the phase transition position (pressure, temperature) accordingly. Vibration experiments can be divided into internal and external vibration experiments according to the relative location of vibration source.

#### 3.3.1. External Vibration

In the external vibration, the cooling medium located around the sample was used as the vibration source, and the sample located at the core served as the target object. The shock vibration generated by the instantaneous volume reduction of water in the vibration source during the phase transition of ice I to ice III was used to realize the synchronous or advance phase transition of ice I to ice III in the target object. In the external vibration test, frozen pure water, frozen 5% sodium chloride solution and frozen 5% glucose solution were used as vibration sources, and frozen apple juice was used as the core target.

**Figure 5:**
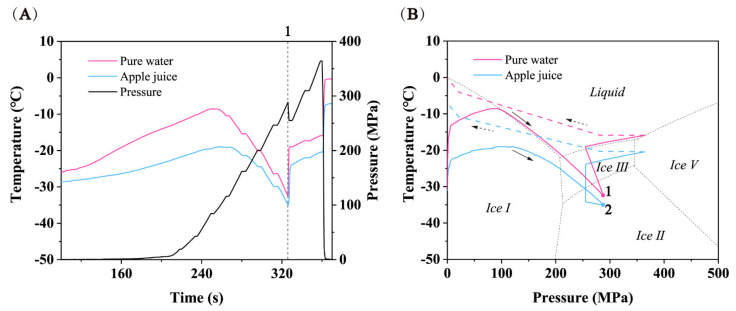
Temperature and pressure paths of frozen inoculated SSPS cooled by 20% sodium chloride solution. (**A**) Temperature and pressure curves over time. (**B**) Temperature-pressure profile superimposed on the phase diagram of water. (—) pressure boost process, (---) pressure relief process.

Taking the temperature and pressure curves of frozen pure water vibrating frozen apple juice as an example (Figure 5), when the pressure rose, the temperature of pure water and apple juice gradually went up due to pressure work and heat exchange with the pressure transmitting medium. The temperature of pure water rose faster than that of internal apple juice (Figure 5A) because the outer pure water was in direct contact with the pressure transmitting medium at room temperature. The temperature of pure water soon reached the phase transition curve of ice I-liquid, and then the temperature began to change along the phase transition curve, which was confirmed by the high coverage of the temperature-pressure curve of pure water and the biphasic line of ice I-liquid in the phase diagram of pure water (Figure 5B).

Because the freezing point of pure water is higher than that of any other solution, when the pure water reached the phase transition curve of ice I-liquid, its temperature curve was placed above the temperature curve of apple juice, which was different from the temperature curve placed below the sample temperature curve when 20% sodium chloride solution was used as a cooling medium in Section 3.1. As the temperature of outside pure water was higher than that of the inside apple juice, the temperature of apple juice gradually increased and slowly reached its own ice I-liquid phase transition curve, thereafter moving along the phase transition curve. When the pressure exceeded 210 MPa, both the pure water and apple juice entered the metastable region of ice III domain and ice II domain successively. When the pressure reached 290 MPa (Figure 5A(1)), the pure water underwent phase transitions from metastable ice I and metastable water to ice III (Figure 5B(1)), while the apple juice underwent phase transitions from metastable ice I and metastable water to ice III simultaneously (Figure 5B(2)). Possible explanations for the synchronous phase transition between pure water and apple juice were as follows: (1) When pure water underwent phase transitions from metastable ice I and metastable water to ice III, the volume decreased instantly. This phenomenon can be verified by the sudden reduction of pressure in the pressure-time curve (Figure 5A(1)), which reduced the pressure from 290 MPa to 255 MPa; (2) At the moment when the volume of pure water decreased, the gap between pure water and apple juice also appeared, so that the pure water was instantly squeezed to fill the gap. In this process, the shock vibration of pure water on the internal apple juice was also generated; (3) Because apple juice was located in the metastable domain of ice III region, the generation of shock vibration just gave apple juice energy to break through the energy barrier of phase transitions from metastable ice I and metastable water to ice III, realizing the synchronous phase transformation of the two. The same phenomenon was observed in repeated experiments.

In the study of pure water as a vibration source and apple juice as the target object, it was observed that shock vibration can affect the phase transition position of ice crystals. In order to further evaluate the impact of the magnitude of the shock vibration (instantaneous reduction of pressure during phase change of vibration source) and the position of the shock vibration (position of the pressure during phase change of vibration source) on the phase change position of the target object, further research was carried out with 5% sodium chloride solution and 5% glucose solution as the vibration source and apple juice as the target object. There were two reasons for choosing these solutions as vibration sources: (1) In the preliminary experiments, it was observed that the phase transition pressures of metastable ice I and metastable water to ice III in 5% sodium chloride solution and 5% glucose solution were 246 MPa and 229 MPa, respectively, which happened earlier than that of pure water, so it can be used as the difference in shock vibration position; (2) During the phase transition of 5% sodium chloride solution and 5% glucose solution, the instantaneous reduction of pressure was 23.8 MPa and 17.8 MPa, respectively, which can be used as the difference of the magnitude of the shock vibration. The results are shown in Table 3.

By comparing the results in Table 3, it can be found that although the phase transition pressure of 5% glucose solution (229 MPa) was relatively forward, the shock vibration generated during phase transition was relatively small (17.8 MPa), and the shock vibration at this time was not large enough to break the metastable limit of apple juice in metastable state. Therefore, apple juice did not realize the early phase transition, and there was no significant difference (*p* > 0.05) in phase transition pressure. On the contrary, although the phase transition pressure (246 MPa) of 5% sodium chloride solution was relatively backward, the shock vibration generated during phase change was relatively large (23.8 MPa), which gave enough energy to the apple juice in metastable state to break through the energy barrier of phase transformation; thus, the phase transition took place in advance, and the phase transition pressure was significantly (*p* < 0.05) advanced by 40 MPa.

To summarize, the impact of the magnitude of the shock vibration of the vibration source on the phase change position of the target object cannot be ignored. Although the position of the shock vibration of the vibration source in front of the target object in this experiment did not necessarily lead to the early phase change of the target object, the impact of the shock vibration position cannot be completely ruled out. Perhaps the phase transition pressure of 5% glucose solution in this experiment was too close to the triple point of liquid-ice I-ice III (210 MPa), and the phase transition reaction near the triple point was more complex, which affected the transmission of shock vibration.

#### 3.3.2. Internal Vibration

In the internal vibration experiments, the sample located inside the cooling system was used as the vibration source, and the cooling medium around the sample was used as the target object. The instantaneous reduction of volume of water in the vibration source during the phase transition of ice I to ice III was used to produce instantaneous collapse vibration in the target object and promoted the ice I to ice III phase transformation in the target object to occur in advance.

In the internal vibration experiment, 5% sodium chloride solution and 5% glucose solution were used as vibration sources, and pure water was used as the target object. The experimental results are shown in Table 4. The phase transition pressure of pure water was obtained in the preliminary experiment and was 292 MPa. Both 5% glucose solution and 5% sodium chloride solution can realize the early phase transition of pure water and advance the phase transition pressure of pure water to 255 MPa and 267 MPa, respectively. Although glucose solution advanced the phase transition pressure of pure water to a greater extent, there was no significant difference (*p* > 0.05) between the phase transition pressure of pure water obtained by glucose solution and sodium chloride solution as a vibration source. According to the standard deviation of phase transition pressure of pure water, it can be found that although the phase transformation of pure water can be realized in advance through an internal vibration test, the position of phase transition was still unstable, which may be caused by the random occurrence of phase transition of water in the vibration source. This phenomenon also existed in external vibration experiments.

The principle of internal vibration experiment can be described as follows: (1) when metastable ice I and metastable water to ice III phase transitions occurred in the vibration source (5% sodium chloride solution and 5% glucose solution), the volume decreased instantaneously; (2) a gap was created between the vibration source at the center of the cooling system and the surrounding target object (pure water). Under the action of high pressure, the pure water collapsed and was squeezed together instantly; (3) at this time, the pure water in metastable state gained energy due to collapse vibration, broke through the energy barrier of phase transition, and completed the phase transition of metastable ice I and metastable water to ice III. The external vibration method has proved that the instantaneous reduction of pressure (the magnitude of shock vibration) during the phase change of vibration source had an impact on the early occurrence of phase change of target object. Hence, in the internal vibration experiment, the sample volume was changed from 4 mL to 50 mL in order to produce greater collapse vibration to realize the early phase transition. The advantage of the internal vibration method is that it improves the processing capacity of the target object from 4 mL of the external vibration method to 400 mL of the internal vibration method, and the amount of vibration source (50 mL) in the internal vibration method is much less than that in the external vibration method (446 mL), which can be of great significance in the cost-savings of a practical application.

These vibration experiments are evaluated as possible contributors for microbial destruction enhancement achieved in the first part of this study when the samples were subjected to HP treatment under fully frozen conditions. Under these conditions, the samples experience phase transition effects and vibrational effects can predominate. This type of detailed analysis of metastable state kinetics and positional effects on microbial destruction has never been analyzed before, and such research is only the beginning. In the future, more detailed experimental evaluation can be expected to be carried out in order to more deeply analyze the law and mechanism of ice crystal phase transition, as well as to carefully evaluate the effect of these vibrations on microbial destruction kinetics.

## 4. Conclusions

The self-cooling equipment developed in our laboratory can maintain test samples in a frozen state even when the pressure chamber is held at room temperature processing conditions, thus enabling the phase transition studies to be carried out without sophisticated equipment. The inactivation of *E. coli* in SPSS was demonstrated to be different when HP treated in unfrozen, frozen-thawed and fully frozen states. The freezing process before compression, the synergistic effect of pressure and freezing during HP treatment and phase transition from ice I to ice III contributed the best inactivation effect, especially the phase transition process. In addition to microbial kinetics, experiments were also designed to understand the vibration behavior of test samples undergoing phase transitions. By selecting the appropriate vibration source, the target object can break through the metastable state in advance and realize the phase transition from ice I to ice III, which was the first attempt in the research field of HPLT phase transition. Factors affecting the phase transition position need to be further explored in the future, and relevant research is also underway. Because the phase change pressure is an important working parameter in the process of phase change microbial, breaking the metastable state and advancing the phase change position means that it can greatly reduce the working pressure during industrialization. Considering the economic cost and equipment safety, this is the best of both worlds.

Current experiments have demonstrated the effect of phase change microbial destruction. The vibration studies have opened up new avenues of research in the phase transition inactivation of microorganisms.

## Figures and Tables

**Table 1:** Experimental design of phase transition position studies.

	Internal Vibration	External Vibration
Sample	S, G	A
Sample volume	50 mL	4 mL
Cooling medium	P	S, G, P
Experiment condition	360 MPa, 0 s	360 MPa, 0 s

S: 5% sodium chloride solution; G: 5% glucose solution; A: apple juice; P: pure water.

**Table 2:** Lethal effects of high-pressure treatment on *E. coli* under three different conditions.

Initial State	Pressure (MPa)	Lethality (log)
Unfrozen	150	0.01 ± 0.01 a
	240	0.25 ± 0.05 a
	330	1.14 ± 0.15 b
Frozen-Thawed	150	1.31 ± 0.10 b,c
	240	1.54 ± 0.07 c,e
	330	2.92 ± 0.12 d
Frozen	150	1.79 ± 0.02 e
	240	2.29 ± 0.37 f
	330	3.68 ± 0.16 g

Different lowercase letters (a–g) represent the significance differences under different treatments (*p* < 0.05); lethal effect = logN_0_ − logN, where N_0_ and N are the initial colony number and the colony number after HP treatment respectively.

**Table 3:** The phase transition pressure of apple juice in external vibration experiment.

Vibration Source	Transient Pressure Reduction	Target Object	Phase Transition Pressure (MPa)
-	-		290 ± 6.2 a
5% glucose solution	17.8 ± 3.8	apple juice	307 ± 15.5 a
5% sodium chloride solution	23.8 ± 2.9		251 ± 9.3 b

Different lowercase letters (a,b) represent the significance differences under different treatments (*p* < 0.05). Transient pressure reduction: the reduction of the volume during the phase transition caused the pressure transient drop, and the transient pressure reduction can be defined as the pressure change caused by the phase change of the vibration source (446 mL), ignoring the change caused by the small volume (4 mL) of apple juice.

**Table 4:** The phase transition pressure of pure water in internal vibration experiment.

Vibration Source	Target Object	Phase Transition Pressure (MPa)
-	pure water	292 ± 4.2 a
5% glucose solution		255 ± 11.9 b
5% sodium chloride solution		267 ± 6.7 b

Different lowercase letters (a,b) represent the significance differences under different treatments (*p* < 0.05).

## Data Availability

Not applicable.
